# Mycobacterial Testing Trends, United States, 2009–2015^1^

**DOI:** 10.3201/eid2609.200749

**Published:** 2020-09

**Authors:** Samantha G. Dean, Emily E. Ricotta, Jonathan Fintzi, Yi Ling Lai, Sameer S. Kadri, Kenneth N. Olivier, Adrian Zelazny, D. Rebecca Prevots

**Affiliations:** National Institutes of Health, Bethesda, Maryland, USA

**Keywords:** nontuberculous mycobacteria, pulmonary disease, epidemiology, tuberculosis and other mycobacteria, bacteria, NTM, acid-fast bacilli, electronic health records, acid-fast bacilli testing, United States

## Abstract

We studied 31 US healthcare facilities to characterize trends in mycobacterial testing. During 2009–2015, testing for acid-fast bacilli increased 3.2% annually, and prevalence of pathogenic nontuberculous mycobacteria increased 4.5% annually. These increases were highest for subpopulations at high risk of infection, including older women, Asians, and patients with concurrent conditions.

Nontuberculous mycobacteria (NTM) are opportunistic environmental pathogens that can cause chronic lung disease (*1*,*2*). NTM are identified through laboratory testing for acid-fast bacilli, which test for all mycobacteria, including *Mycobacterium tuberculosis*. Multiple studies have described increasing NTM pulmonary disease (NTM PD) prevalence in the United States (*1*,*3*–*6*), a phenomenon that might be caused by true increase in disease rates, new efficient testing technologies, increased mycobacterial testing, or any combination of those. We assessed trends in mycobacterial testing rates and NTM PD prevalence from 2009 through 2015. We also analyzed factors associated with differential testing rates and prevalence across subpopulations.

## The Study

The population for our study comprised persons whose medical encounters were represented in the Cerner HealthFacts Electronic Health Record database (https://sc-ctsi.org/resources/cerner-health-facts). We extracted microbiological, demographic, and clinical data for all patient encounters at 31 facilities across the United States that continually reported microbiological data (Appendix) and that speciated mycobacterial culture results from 2009 through 2015. We included only microbiology data collected from pulmonary body sites and that used the words “AFB” and “culture” in the testing description (i.e., mycobacterial culture tests). For analyses of mycobacterial culture testing and pathogenic NTM culture positivity rates (Appendix), we used the number of unique inpatients and outpatients at the 31 facilities as the population denominator.

To estimate mycobacterial culture testing trends, we used Poisson regression models fit through quasi-likelihood methods, which enable overdispersion (*7*). We analyzed trends within the overall study population and subpopulations stratified by age, sex, race/ethnicity, concurrent conditions, facility size, region, and facility teaching status. To identify variables associated with the odds of mycobacterial culture testing per facility encounter and the odds of pathogenic NTM culture positivity per facility encounter, we fit 2 mixed-effect logistic regression models to the data. We adjusted these models for patient age, sex, interactions between age and sex, race/ethnicity, teaching facility status, facility census region, encounter year, and whether the patient had a pulmonary computed tomographic scan or radiograph during the study period. The following concurrent conditions have been associated with a higher risk for NTM PD and were included as predictors in the model: bronchiectasis (*4*,*8*), chronic obstructive pulmonary disease (*4*,*8*), cystic fibrosis (CF) (*9*), lung cancer (*4*,*5*,*8*), and rheumatoid arthritis (*8*). We included deidentified patient number as a random effect to account for clustering among an individual patient’s multiple encounters.

Persons with mycobacterial culture tests were older and had more concurrent conditions than the overall population in the 31 study facilities: 20,670 (43%) of 48,563 persons with mycobacterial cultures were >65 years of age, compared with 1,984,443 (18%) of 10,802,134 persons in the overall study population (Table 1). Patients with the stated pulmonary conditions had higher rates of testing and NTM positivity than the overall study population. Bronchiectasis patients had mycobacterial culture tests (1,832/10,000 patients) and tested positive for NTM (339/10,000 patients) at higher rates than any analyzed subpopulation (other than persons with CF). Although patients who identified as Asian were tested at a lower rate than patients who identified as White or African American (48/10,000 patients), their positivity rate of 5.6 per 10,000 patients was the highest of the 3 racial/ethnic groups examined in this study (Table 1).

**Table 1:** Rates of laboratory testing for AFB and pathogenic NTM positivity, United States, 2009–2015*

Variable		No. (%) patients†	No. (%) patients tested for AFB	Tests for AFB/10,000 patients‡	Pathogenic NTM cases/10,000 patients
Total		10,802,134 (100.0)	48,563 (100.0)	45.0	3.1
Sex					
F		5,599,841 (51.8)	22,975 (47.3)	41.0	3.0
M		4,545,803 (42.1)	25,585 (52.7)	56.3	3.6
Age, y					
<65		9,041,231 (83.7)	27,830 (57.3)	30.8	1.9
≥65		1,984,443 (18.4)	20,670 (42.6)	104.2	8.3
Sex and age, y					
F, <65		4,638,813 (42.9)	12,797 (26.4)	27.9	1.6
F, ≥65		1,089,079 (10.1)	10,144 (20.9)	94.6	8.8
M, <65		3,817,761 (35.3)	15,030 (30.9)	39.8	2.5
M, ≥65		816,161 (7.6)	10,526 (21.7)	131.0	8.6
Census region					
Midwest		2,112,964 (19.6)	11,866 (24.4)	56.2	4.7
Northeast		4,155,756 (38.5)	16,203 (33.4)	39.0	2.2
South		3,020,093 (28.0)	14,823 (30.5)	49.1	3.1
West		1,513,321 (14.0)	5,671 (11.7)	37.5	3.2
Race§					
African American		1,645,676 (15.2)	8,639 (17.8)	52.5	3.4
Asian		306,103 (2.8)	1,458 (3.0)	47.6	5.6
White		6,411,413 (59.4)	34,300 (70.6)	53.5	3.6
Concurrent conditions					
Lung cancer		56,719 (0.5)	3,729 (7.7)	657.5	24.9
Rheumatoid arthritis		52,004 (0.5)	711 (1.5)	136.7	6.7
Cystic fibrosis		3,835 (0.04)	865 (1.8)	2,255.5	276.4
Chronic obstructive pulmonary disease		165,107 (1.5)	4,301 (8.9)	260.5	19.9
Bronchiectasis		8,666 (0.1)	1,588 (3.3)	1,832.4	339.3
Teaching status indicator¶					
Nonteaching		2,094,368 (19.4)	7,815 (16.1)	37.3	3.3
Teaching		8,816,749 (81.6)	39,592 (81.5)	44.9	3.0

*AFB, acid-fast bacilli; NTM, nontuberculous mycobacteria. †Stratified totals do not always add up to 100% because of missing data and patients’ membership in multiple categories. ‡Patients with multiple tests or positive isolates are counted a single time. §Racial/ethnic groups with small sample sizes and patients categorized as “Unknown” are not shown in stratified analysis. ¶Teaching status indicator refers to whether a facility visited by a patient is a teaching facility.

**Figure 1:**
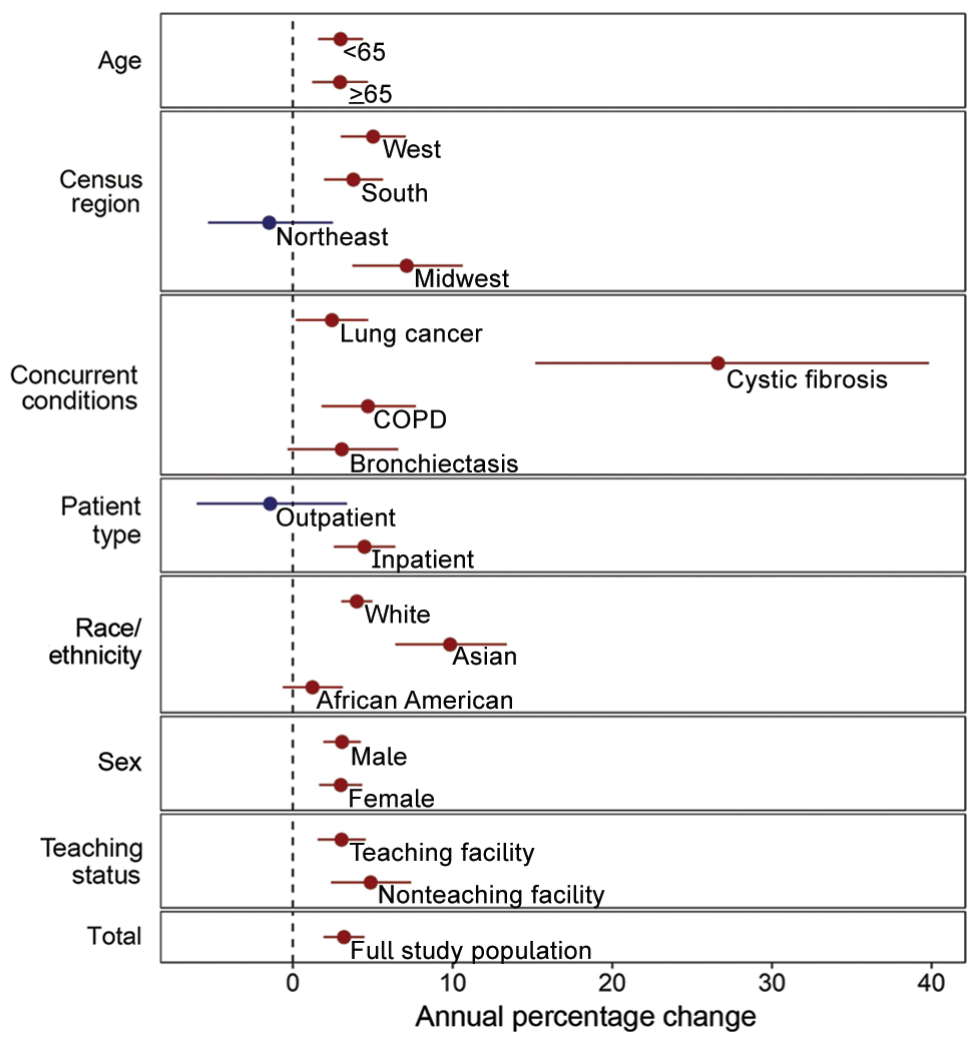
Annual percentage change in laboratory testing for acid-fast bacilli in 31 facilities, United States, 2009–2015. Red indicates increasing trends; blue indicates decreasing trends. Error bars indicate 95% CI. COPD, chronic obstructive pulmonary disease.

**Figure 2:**
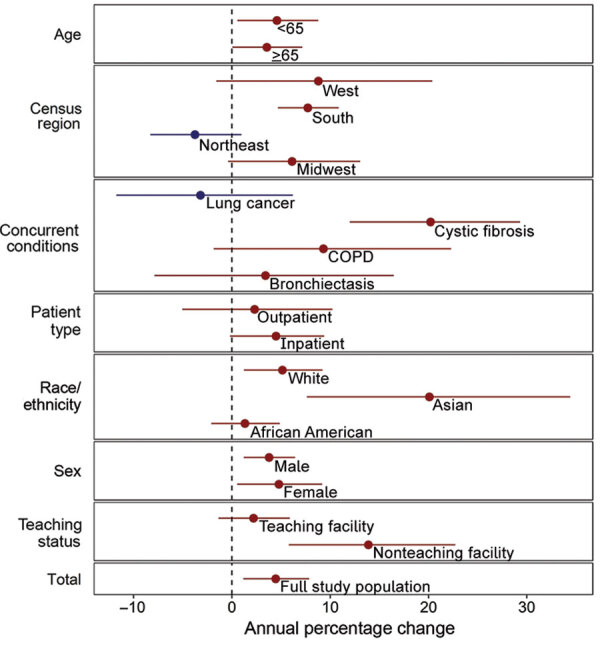
Annual percentage change in identified pathogenic nontuberculous mycobacteria (NTM) in 31 facilities, United States, 2009–2015. Red indicates increasing trends; blue indicates decreasing trends. Error bars indicate 95% CI. COPD, chronic obstructive pulmonary disease.

From 2009 through 2015, the average annual increase in mycobacterial culture testing was 3.2% per year (95% CI 1.9%–4.5%) across all facilities. The average annual increase in pathogenic NTM positivity was 4.5% per year (95% CI 1.2%–7.9%) (Appendix Figure). Across subgroups, point estimates consistently showed an increase in testing and positivity (Figures 1, 2). Testing and positivity rates increased at a higher rate among persons who identified as Asian than among other racial/ethnic groups; among Asians, rates of culture testing increased 9.8% per year (95% CI 6.4%–13.4%), and culture positivity increased 20.1% per year (95% CI 7.6%–34.4%). Among persons with CF, rates of testing increased 26.6% per year (95% CI 15.2%–39.8%), and positivity increased 20.2% per year (95% CI 12.0%–29.3%). We observed decreasing trends in testing and positivity for patients in the Northeast census region; however, these trends were not significant (Figures 1, 2).

Using multivariable analysis we found male sex, Asian race/ethnicity, older age, concurrent pulmonary conditions, and admission to teaching facilities to be positively associated with mycobacterial culture testing and pathogenic NTM culture positivity. Encounters of women >65 years of age had 2.1-fold (95% CI 2.1–2.2) higher odds of mycobacterial culture testing compared with those for women <65 years of age. Encounters of persons who identified as Asian had 1.8-fold (95% CI 1.7–1.9) higher odds of mycobacterial culture testing compared with encounters of those who identified as White. All selected concurrent conditions were associated with increased odds of receiving a mycobacterial culture test. The highest odds were for persons with CF or bronchiectasis: compared with persons without these conditions, the odds of testing increased 18.4-fold (95% CI 16.6–20.3) for those with CF and 6.7-fold (95% CI 6.3–7.2) for those with bronchiectasis (Table 2).

**Table 2:** Mixed-effect logistic regressions predicting laboratory testing for acid-fast bacilli and positivity for pathogenic nontuberculous mycobacteria, United States, 2009–2015*

Variable†	Testing for acid-fast bacilli, OR (95% CI)	Pathogenic nontuberculous mycobacteria positivity, OR (95% CI)
Sex‡		
M, age >65 y; ref: F, >65 y	1.5 (1.4–1.5)	1.1 (0.9–1.3)
M, age <65 y; ref: F, <65 y	1.7 (1.7–1.8)	1.8 (1.5–2.1)
Sex and age, y		
Age >65 y, M; ref: M, age <65 y	1.8 (1.7–1.8)	1.9 (1.6–2.3)
Age >65 y, F; ref: F, age <65 y	2.1 (2.1–2.2)	3.2 (2.7–3.8)
Race/ethnicity		
Asian; ref: white	1.8 (1.7–1.9)	2.6 (2.0–3.5)
African American; ref: white	1.0 (1.0–1.0)	1.0 (0.9–1.2)
Hispanic; ref: white	0.8 (0.7–0.8)	1.1 (0.7–1.6)
Concurrent conditions§		
Bronchiectasis	6.7 (6.3–7.2)	3.0 (2.5–3.6)
Chronic obstructive pulmonary disease	2.7 (2.6–2.8)	1.8 (1.6–2.1)
Cystic fibrosis	18.4 (16.6–20.3)	7.7 (4.7–12.5)
Lung cancer	4.5 (4.3–4.7)	1.3 (1.0–1.7)
Rheumatoid arthritis	1.4 (1.3–1.5)	0.7 (0.5–1.1)
Pulmonary computed tomographic scan or radiograph	3.3 (3.1–3.5)	1.5 (1.1–2.2)
Teaching facility; ref: nonteaching facility	1.6 (1.5–1.6)	1.4 (1.2–1.7)
Region		
Midwest; ref: Northeast	1.1 (1.1–1.1)	1.5 (1.3–1.8)
South; ref: Northeast	1.7 (1.6–1.7)	1.7 (1.4–2.0)
West; ref: Northeast	1.7 (1.7–1.8)	2 (1.5–2.5)

*OR, odds ratio; ref, referent. †Model also adjusted for year as a potential confounder. ‡Age and sex odds ratios calculated with interaction term. §Concurrent conditions were ascertained by codes from the International Classification of Diseases, 9th and 10th Revision. Computed tomographic scans and radiographs were identified through text searching procedure descriptions.

Encounters of women >65 years of age had 3.2-fold (95% CI 2.7–3.8) higher odds of NTM positivity compared with those for women <65 years of age. Persons who identified as Asian had 2.5-fold (95% CI 1.8–3.4) higher odds of culture positivity compared with those for persons who identified as white. Concurrent conditions increased the odds of testing 7.7-fold (95% CI 4.7–12.5) for patients with CF and 3.0-fold (95% CI 2.5–3.6) for patients with bronchiectasis (Table 2).

## Conclusions

An important feature of our study is the analysis of both mycobacterial culture testing and NTM positivity data in a single population. We found that mycobacterial culture testing increased at an average of 3.2% per year (95% CI 1.9%–4.5%), whereas pathogenic NTM culture positivity increased an average of 4.5% per year (95% CI 1.2%–7.9%). This finding builds on previous work identifying an increase in prevalence of NTM PD (*1*,*3*–*6*). Increased testing might facilitate case identification and therefore might contribute to increasing NTM PD prevalence. Continued testing, particularly among populations at high risk, could advance understanding of NTM PD prevalence for improved clinical and public health planning.

Our analysis is consistent with prior studies showing the highest NTM PD prevalence among older women, Asians, and persons with CF, bronchiectasis, and chronic obstructive pulmonary disease (*1*). Our estimate of a 4.5% (95% CI 1.2%–7.9%) annual increase in NTM culture positivity is comparable with an estimated 7.5% (95% CI 6.7%–8.2%) annual increase in NTM PD prevalence from 2008 to 2015 made using a large managed care claims database (*6*). Further, many identified predictors of receiving a mycobacterial culture test are consistent with predictors of positivity, as identified in this and previous studies. This finding suggests that tests are being successfully focused toward groups such as older women and Asians that are at high risk for culture positivity (*1*).

Mycobacterial culture testing might be increasing because of greater awareness of NTM PD among groups at high risk. In 2012, the Cystic Fibrosis Foundation published guidelines recommending that CF patients be cultured annually for NTM (*10*). Furthermore, numerous studies published since 2010 have linked NTM PD to other concurrent conditions (*4*,*8*,*9*,*11*). These findings might contribute to improved awareness and increased testing, especially in populations at high risk for NTM PD.

Appendix Additional information on methods and facility characteristics.
