# Supplementary material for: Mycobacterial Testing Trends, United States, 2009–2015
Source: Emerg Infect Dis. 2020 Sep;26(9):2243–6. doi: 10.3201/eid2609.200749 (PMC7454078; doi:10.3201/eid2609.200749)
Supplement: Appendix — Additional information on methods and facility characteristics. [file 20-0749-Techapp-s1.pdf]

Article DOI: https://doi.org/10.3201/eid2609.200749

Mycobacterial Testing Trends, United States, 2009–2015

Appendix

Extended Methods

Mycobacteria were considered pathogenic if they were clinically significant species other than *Mycobacterium tuberculosis*. *Mycobacterium gordonae* were excluded. Continuously reporting hospitals were defined as those for which the number of mycobacterial tests did not decrease more than 40% between consecutive years and at least 10 *Mycobacterium avium* complex (MAC)-associated cases were reported during the study period. We chose this approach because MAC is the predominant NTM PD species in the United States (*1*), and we wanted to ensure that we included only facilities where species identification results were captured by the electronic health record.

Trend analysis was conducted in a series of univariate Poisson regressions fit via quasi-likelihood, referred to as quasi-Poisson models. For models estimating mycobacterial testing trends, the dependent variable was the number of mycobacterial culture tests in a year. For models estimating pathogenic NTM positivity, the dependent variable was the number of positive cultures in a year. All models included year as the independent variable and the natural log of the population size of the 31 study hospitals as the offset. We did not adjust for multiplicity. The small sample sizes for the subpopulations of patients with pulmonary computed tomography scans / x-rays or rheumatoid arthritis were insufficient to include in trend analysis.

Extended Discussion

Among patients aged ≥65 years, mycobacterial culture testing and NTM positivity were present at a higher rate in women than men. Among patients aged <65 years, testing and NTM positivity were present at a higher rate in men than women (Table 1). A possible explanation for this is the higher prevalence of certain comorbidities among men aged <65 years compared to

women aged <65 years in our study population. Men aged <65 years have COPD at a rate of 92 cases per 10,000 patients, and women aged <65 years have COPD at a rate of 81 cases per 10,000 patients. Men aged <65 years have lung cancer at a rate of 31 cases per 10,000 patients while women aged <65 years have lung cancer at a rate of 27 cases per 10,000 patients.

To assess generalizability of our study, we compared the characteristics of the included Cerner facilities to the 2014 American Hospital Association's database of hospitals (*2*) (Appendix Table). Selecting for hospitals that report detailed microbiology changes the distribution of characteristics compared to the full Cerner database. The facilities in our study are more likely to be larger (more hospital beds), teaching facilities, and located in the Northeast. However, our study has a robust representation of high-risk groups, particularly those with bronchiectasis, chronic obstructive pulmonary disease, cystic fibrosis, as well as older adults and persons identifying as Asian, and these population are of greatest interest for assessing trends in mycobacterial testing and NTM prevalence.

A Note on Tuberculosis

To explore the possibility that increases in testing have been driven by tuberculosis (TB) trends, we analyzed the number of patients with International Classification of Diseases, 9th and 10th revision, codes for suspected TB and the number of cultures positive for TB. In the full population of the 31 study hospitals, 453 persons received an ICD9/10 code for suspected TB from 2009 through 2015. Of these 453 persons, 37 received a mycobacterial culture test. The rarity of this diagnosis suggests that the observed increase in testing is not driven by increasing suspicion of TB. Similarly, we looked at rates of isolated M. tuberculosis. There were 503 persons with isolated M. tuberculosis among the 48,563 persons who received a mycobacterial test. The annual prevalence of M. tuberculosis isolates was not increasing significantly, with an annual percent change of 1.2% (95% CI: –5.4 to 8.4). The lack of evidence for a significant increase in TB supports our assumption that testing trends are not fully driven by trends in TB.

Appendix Table. Comparison of Cerner study facilities and American Hospital Association (AHA) facilities, United States, 2009–2015

Variable	No. Cerner study facilities (%), N = 31	No. AHA facilities (%),* N = 6,174
Region		
Midwest	6 (19)	1,704 (28)
Northeast	13 (42)	804 (13)
South	8 (26)	2,506 (41)
West	4 (13)	1,160 (19)
Setting		
Urban	29 (94)	4,998 (81)
Rural	2 (6)	1,176 (19)
Teaching status		
Nonteaching	7 (23)	4,025 (65)
Teaching	23 (74)	2,149 (35)
Facility bed size		
0–99	2 (6)	3,414 (55)
100–199	2 (6)	1,241 (20)
200–299	13 (42)	630 (10)
300–499	7 (23)	578 (9)
≥500	7 (23)	311 (5)

*AHA data adapted from (*2*).

References

1. Adjemian J, Daniel-Wayman S, Ricotta E, Prevots DR. Epidemiology of nontuberculous mycobacteriosis. Semin Respir Crit Care Med. 2018;39:325–35. PubMed https://doi.org/10.1055/s-0038-1651491

2. Balk RA, Kadri SS, Cao Z, Robinson SB, Lipkin C, Bozzette SA. Effect of procalcitonin testing on health-care utilization and costs in critically ill patients in the United States. Chest. 2017;151:23–33. PubMed https://doi.org/10.1016/j.chest.2016.06.046

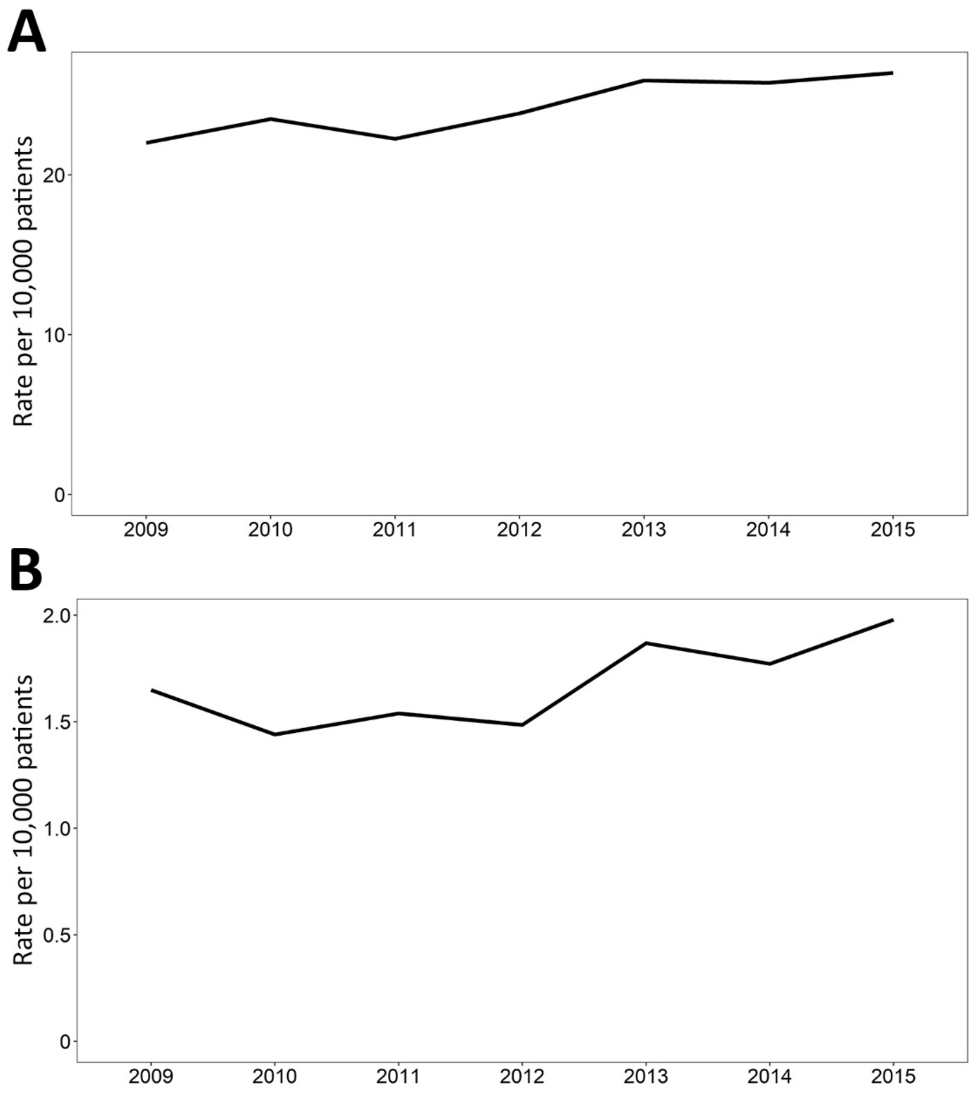

Appendix Figure. Trends in laboratory testing for acid-fast bacilli (A) and pathogenic nontuberculous mycobacteria positivity (B) in 31 US hospitals, 2009–2015
